# Supplementary material for: A UMLS-based spell checker for natural language processing in vaccine safety
Source: BMC Med Inform Decis Mak. 2007 Feb 12;7:3. doi: 10.1186/1472-6947-7-3 (PMC1805499; doi:10.1186/1472-6947-7-3)
Supplement: Additional file 13 — RAP application directory. Sets up directory for RAP (RDF application for PHP) source code [file 1472-6947-7-3-S13.gz › rap/doc/phpdoc/__filesource/fsource_dataset__datasetIteratorFindQuadsMem.php.html]

xml version="1.0" encoding="iso-8859-1"?


File Source for IteratorFindQuadsMem.php


# Source for file IteratorFindQuadsMem.php

Documentation is available at IteratorFindQuadsMem.php

```
1. <?php
2. /**
3. * ----------------------------------------------------------------------------------
4. * Class: IteratorFindQuadsMem
5. * ----------------------------------------------------------------------------------
6. *
7. * @package     dataset
8. */


11. /**
12. * Implementation of a quad iterator.
13. *
14. * This Iterator should be used like:
15. * for($iterator = $dataset->findInNamedGraphs(null,null,null,null); $iterator->valid(); $iterator->next())
16. * {
17. *    $currentQuad=$it->current();
18. * };
19. *
20. * <BR><BR>History:
21. * <LI>05-11-2005                : First version of this class.</LI>
22. *
23. * @version  V0.9.1
24. * @author Daniel Westphal (http://d-westphal.de)
25. *
26. *
27. * @package     dataset
28. * @access    public
29. ***/
30. class IteratorFindQuadsMem
31. {
32. /**
33. * key value in the current graph
34. * @var     dataset
35. * @access    private
36. */
37. var $graphKey;

39. /**
40. * boolean value, if the results should be returned as triples
41. * @var        boolean
42. * @access    private
43. */
44. var $returnAsTriples;

46. /**
47. * The current position
48. * @var        integer
49. * @access    private
50. */
51. var $key;

53. /**
54. * If the current resource is valid
55. * @var        boolean
56. * @access    private
57. */
58. var $valid;

60. /**
61. * The current NamedGraph
62. * @var  NamedGraph
63. * @access    private
64. */
65. var $current;

67. /**
68. * The graphName Resource to search for
69. * @var string
70. * @access    private
71. */
72. var $findGraphName;

74. /**
75. * The subject Resource to search for
76. * @var string
77. * @access    private
78. */
79. var $findSubject;

81. /**
82. * The predicate Resource to search for
83. * @var string
84. * @access    private
85. */
86. var $findPredicate;

88. /**
89. * The object Resource to search for
90. * @var string
91. * @access    private
92. */
93. var $findObject;

95. /**
96. * Iterator over all graphs of the RDF dataset
97. * @var string
98. * @access    private
99. */
100. var $graphIterator;


103. /**
104. * Constructor.
105. *
106. * $subject, $predicate, and $object are used like find().
107. * $getSPO supports the strings 's', 'p', and 'o' to return
108. * either the subject, predicate, or object of the result statements.
109. *
110. *
111. * @param Resource
112. * @param Resource
113. * @param Resource
114. * @param dataset
115. * @param Boolean
116. * @access    public
117. */
118. function IteratorFindQuadsMem($subject,$predicate,$object,&$graphIterator, $returnAsTriples=false)
119. {
120. $this->findSubject=$subject;
121. $this->findPredicate=$predicate;
122. $this->findObject=$object;
123. $this->graphIterator=&$graphIterator;
124. $this->rewind();
125. $this->returnAsTriples=$returnAsTriples;
126. }

128. /**
129. * Resets iterator list to start
130. *
131. * @access    public
132. */
133. function rewind()
134. {
135. $this->graphIterator->rewind();
136. $this->key = -1;
137. $this->graphKey=-1;
138. $this->next();
139. }

141. /**
142. * Says if there are additional items left in the list
143. *
144. * @return    boolean
145. * @access    public
146. */
147. function valid()
148. {
149. return $this->valid;
150. }

152. /**
153. * Moves Iterator to the next item in the list
154. *
155. * @access    public
156. */
157. function next()
158. {
159. if($this->graphIterator->valid()===false)
160. {
161. $this->valid=false;
162. return;
163. }

165. $currentGraph=&$this->graphIterator->current();
166. $this->current= $currentGraph->findFirstMatchingStatement($this->findSubject,$this->findPredicate,$this->findObject,++$this->graphKey);
167. if($this->current==null)
168. {
169. do
170. {
171. $this->graphIterator->next();
172. if($this->graphIterator->valid()===false)
173. {
174. $this->valid=false;
175. return;
176. }
177. $currentGraph=&$this->graphIterator->current();
178. $this->graphKey=-1;
179. $this->current= $currentGraph->findFirstMatchingStatement($this->findSubject,$this->findPredicate,$this->findObject,++$this->graphKey);

181. } while ($this->current==null);
182. }
183. $this->key++;
184. $this->valid=true;
185. }

187. /**
188. * Returns the current item
189. *
190. * @return    mixed
191. * @access    public
192. */
193. function current()
194. {
195. if($this->returnAsTriples) return $this->current;

197. $currentGraph=&$this->graphIterator->current();
198. return new Quad(new Resource($currentGraph->getGraphName()),$this->current->getSubject(),$this->current->getPredicate(),$this->current->getObject());
199. }

201. /**
202. * Returns the key of the current item
203. *
204. * @return    integer
205. * @access    public
206. */
207. function key()
208. {
209. return $this->key;
210. }
211. }
212. ?>
```

Documentation generated on Thu, 7 Jul 2005 13:41:49 +0200 by phpDocumentor 1.3.0RC3
